# Supplementary material for: Two Ligand-Binding Sites on SARS-CoV-2 Non-Structural Protein 1 Revealed by Fragment-Based X-ray Screening
Source: Int J Mol Sci. 2022 Oct 18;23(20):12448. doi: 10.3390/ijms232012448 (PMC9604401; doi:10.3390/ijms232012448)
Supplement: Supplementary file 1 [file ijms-23-12448-s001.zip › ijms-1930239-supplementary.pdf]

Two Ligand-Binding Sites on SARS-CoV-2 Non-Structural Protein 1 Revealed by Fragment-Based X-ray Screening

Shumeng Ma[1], Shymaa Damfo[1], Jiaqi Lou[1], Nikos Pinotsis[2], Matthew W. Bowler[3], Shozeb Haider[1,4] and Frank Kozielski[1]

[1]School of Pharmacy, University College London, 29-39 Brunswick Square, London WC1N 1AX, UK
[2]Institute of Structural and Molecular Biology, Birkbeck College, London WC1E 7HX, UK
[3]European Molecular Biology Laboratory, 38000 Grenoble, France
[4]UCL Centre for Advanced Research Computing, University College London, London WC1H 9RN, UK

Content

Table S1. Crystal images with crystallisation conditions 2

Table S2. Data collection and refinement statistics for SARS-CoV-2 $nsp1_{10-126}$-fragment complexes discussed in this manuscript 3

Table S1: Representative crystallisation conditions and images of three types of SARS-CoV-2 nsp1$_{10\text{-}126}$ crystals. All crystals grew at 18°C at protein concentrations between 15 and 27 mg/mL.

<table>
<tr><th>Commercial screens</th><th>Crystallisation condition</th><th>Crystal images</th></tr>
<tr><td>Index™</td><td>0.1 HEPES pH 7.5, 25% w/v PEG3350</td><td rowspan="3">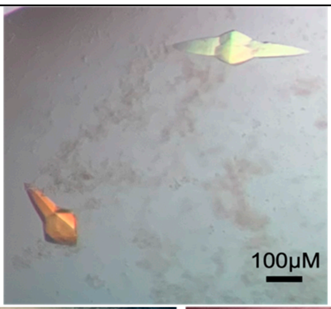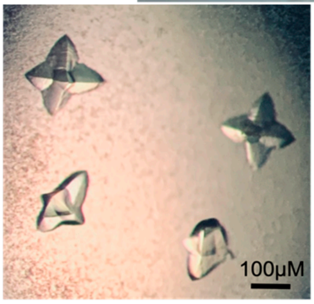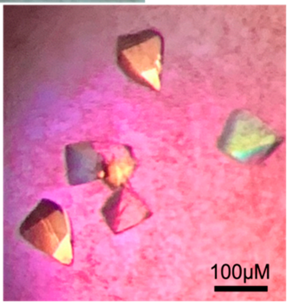</td></tr>
<tr><td>Morpheus®</td><td>0.12 M Ethylene glycol, 0.1 M Imidazole, 0.1 M MES monohydrate acid pH 6.5, 20% v/v Ethylene glycol, 10% w/v PEG8000</td></tr>
<tr><td>JCSG-plus™</td><td>0.2 M Ammonium formate, 20% w/v PEG3350</td></tr>
</table>

Table S2. Data collection, data processing, and model refinement statistics for five nsp1$_{10-126}$-fragment complexes from SARS-CoV-2. Data in parenthesis correspond to the highest resolution shell.

	**Nsp1-11C6**	**Nsp1-10B6**	**Nsp1-5E11**
**PDB ID**	8AZ9	8AYS	8ASQ
**Wavelength [Å]**	0.9655	0.9655	0.9655
**Resolution range [Å]**	35.63 - 1.42 (1.471 - 1.42)	35.51 - 1.37 (1.419 - 1.37)	32.58 - 1.15 (1.191 - 1.15)
**Space group**	$P4_32_12$	$P4_32_12$	$P4_32_12$
**Unit cell parameters (Å, °)**	*a*=*b*=36.817, *c*=141.205, α=β=γ=90	*a*=*b*=36.6882, *c*=140.98, α=β=γ=90	*a*=*b*=36.705, *c*=141.491, α=β=γ=90
**Total reflections**	136107 (14061)	192589 (3956)	313412 (27576)
**Unique reflections**	19225 (1872)	19842 (1315)	35543 (3461)
**Multiplicity**	7.1 (7.5)	9.7 (3.0)	8.8 (8.0)
**Completeness [%]**	99.46 (99.57)	93.33 (63.87)	96.69 (100.00)
**Mean I/sigma(I)**	20.46 (2.85)	23.83 (1.21)	9.77 (0.95)
**Wilson B-factor ($Å^2$)**	18.20	13.88	14.28
**R-meas [%]**	5.202 (67.27)	5.332 (19.74)	9.982 (131.60)
**$CC_{1/2}$**	0.999 (0.838)	0.999 (0.938)	0.997 (0.560)
**R_{cryst}/R_{free} [%]**	15.04 (20.81) / 14.67 (18.00)	14.55 (17.30) / 15.53 (16.06)	20.16 (30.74) / 25.19 (34.72)
**Total no. of non-hydrogen atoms (protein)**	1094	1082	960
**No. of protein/ligand/solvent atoms**	959 / 26/ 120	941 / 21/ 128	876 / 28/ 70
**Average B-factor/protein/ligands/solvent**	25.90 / 23.66 / 82.69 / 37.31	20.86 / 18.85 / 46.03 / 33.09	23.93 / 22.61 / 60.46 / 33.04
**RMSD (bonds, angles)**	0.012 / 1.33	0.012 / 1.26	0.012 / 1.34
**Ramachandran favoured/allowed/outliers/rotamer outliers [%]**	97.32 / 2.68 / 0.00 / 1.87	97.32 / 2.68 / 0.00 / 0.00	99.10 / 0.90 / 0.00 / 1.06
**Clashscore**	7.63	9.36	3.92

Continuation Table S2. Data collection, data processing, and model refinement statistics for five nsp1$_{10-126}$-fragment complexes from SARS-CoV-2. Data in parenthesis correspond to the highest resolution shell.

	**Nsp1-8E6**	**Nsp1-7H2**
**PDB ID**	8AZ8	8AYW
**Wavelength [Å]**	0.9655	0.9655
**Resolution range [Å]**	35.34 - 1.18 (1.222 - 1.18)	35.5 - 1.10 (1.139 - 1.10)
**Space group**	$P4_32_12$	$P4_32_12$
**Unit cell parameters (Å, °)**	a=b=36.643, c=141.366, α=β=γ=90	a=b=36.524, c=141.988, α=β=γ=90
**Total reflections**	297661 (30739)	458335 (28515)
**Unique reflections**	30502 (3207)	40253 (3891)
**Multiplicity**	9.8 (9.6)	11.4 (7.3)
**Completeness [%]**	92.79 (100.00)	99.89 (99.01)
**Mean I/sigma(I)**	18.77 (2.19)	15.90 (1.32)
**Wilson B-factor (Å^2)**	15.64	13.51
**R-meas [%]**	5.976 (97.11)	7.452 (130.9)
**$CC_{1/2}$**	0.998 (0.795)	0.998 (0.589)
**R_{cryst}/R_{free} [%]**	16.65 (19.89) / 20.81 (25.73)	17.04 (26.77) / 19.09 (27.97)
**Total no. of non-hydrogen atoms (protein)**	970	1032
**No. of protein/ligand/solvent atoms**	878 / 24 / 81	919 / 20 / 103
**Average B-factor/protein/ligands/solvent**	24.22 / 22.96 / 54.47 / 33.70	23.42 / 22.01 / 56.28 / 32.75
**RMSD (bonds, angles)**	0.012 / 1.40	0.012 / 1.36
**Ramachandran favoured/allowed/outliers/rotamer outliers [%]**	96.49 / 3.51 / 0.0 / 0.0	94.74 / 5.26 / 0.0 / 1.0
**Clashscore**	1.69	5.89
